# Compositional variation in eye-infiltrating immune cells distinguishes human uveitis subtypes

**DOI:** 10.1016/j.isci.2025.111928

**Published:** 2025-01-30

**Authors:** Christian Concepcion, Yu Xia, Yulia Korshunova, Gregory W. Bligard, Amal Taylor, Michael A. Paley, Philip A. Ruzycki, Lynn M. Hassman

**Affiliations:** 1 Department of Ophthalmology, University of Colorado, Aurora, CO 80045, USA; 2 Department of Ophthalmology and Visual Sciences, Washington University in St. Louis, St. Louis, MO 63108, USA; 3 Department of Medicine, Washington University in St. Louis, St. Louis, MO 63108, USA

**Keywords:** Cell biology, Immunity, Transcriptomics

## Abstract

Uveitis is a heterogeneous group of ocular inflammatory diseases that respond inadequately to empiric therapy, underscoring the need to define pathophysiologic subtypes. Toward this goal, we applied single-cell transcriptional and immune receptor profiling to ocular fluid biopsies from 23 patients with diverse clinical features. We found that a subset of ocular T cells undergoes local clonal expansion, indicating that antigens trigger uveitis in some patients. Infiltrates from HLA-B27 associated acute anterior uveitis (HLA-B27 AAU) were enriched in myeloid cells and innate immune signaling pathways, while chronic uveitis featured prominent adaptive immune cells and transcriptional programs, indicating that immunologic features distinguish uveitis subtypes. Clinical features including anatomic involvement were associated with additional variability in the immune cell profile. These insights highlight the potential for deep transcriptional profiling of ocular immune cells to reveal endotypes within uveitis.

## Highlights

• Immune cell profiling reveals the landscape of ocular inflammation in uveitis
• Tissue enrichment and gene expression distinguishes locally expanded T cells
• Key cell types define immunologic endotypes in uveitis

## Graphical abstract

**Figure:**
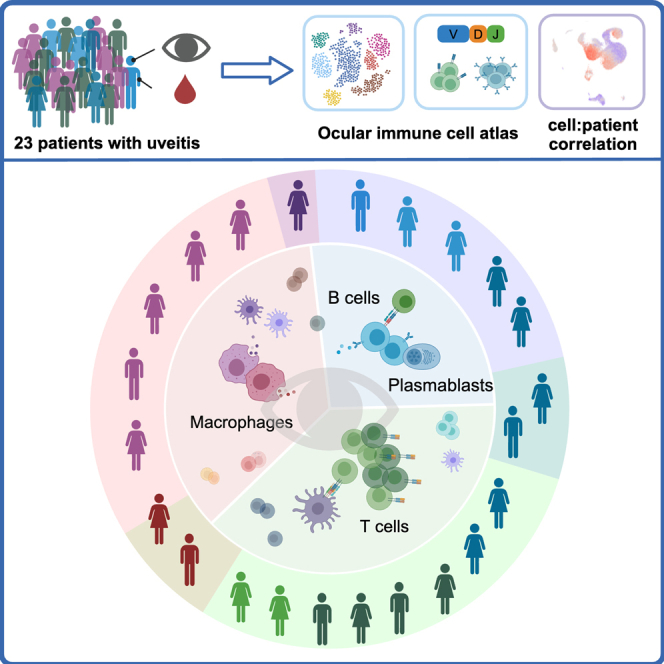

## Introduction

Uveitis is a heterogeneous group of vision-threatening inflammatory diseases that occur within the intraocular space. Currently, these diseases are classified based on clinical features^1^ and few diagnostic markers, such as specific major histocompatibility alleles, are available to aid in disease classification. Common therapies include non-steroidal anti-inflammatory drugs (NSAIDs), steroids, and steroid-sparing immunosuppressive therapies, which incur risk for side effects, such as cataract, increased intraocular pressure, and infections.^2^ Immune therapies are currently applied empirically and patients with similar clinical phenotypes often respond differently to the same therapy, indicating a profound and poorly understood heterogeneity of disease mechanisms. Moreover, many uveitis cases respond poorly to current therapies, highlighting the need for a better understanding of the mechanisms driving ocular inflammation in uveitis.

Currently, uveitis cases are classified based on clinical phenotyping and association with systemic disease,^1^ yet in the absence of extraocular disease or an active infection, most cases of uveitis remain undifferentiated. Acute anterior uveitis (AAU) is characterized by rapid onset of severe ocular pain, redness, loss of vision, and dense accumulations of inflammatory cells and fibrin inside the anterior chamber of the eye. Approximately half of patients with AAU express HLA-B27^3^ and half of these cases are associated with HLA-B27-associated ankylosing spondylitis.^4^ In contrast, patients with chronic inflammation in the anterior chamber usually present without pain and with a slower decline in their vision. Some cases feature large cellular aggregates commonly associated with biopsy-proven granulomas in other tissues, such as sarcoidosis or tuberculosis; in these cases, the descriptor “granulomatous” is often applied. Histopathology of granulomatous uveitis reveals lesions that are similar to granulomas in other tissues,^5^ although, because biopsy of the eye results in significant morbidity, it is not routinely performed.

The intraocular space is relatively sequestered from the rest of the body, separated from the intravascular space by the blood ocular barrier, which excludes most peripheral immune cells at steady state and creates relative immune-privilege analogous to the central nervous system.^6^^,^^7^ Like the central nervous system, which is bathed by cerebrospinal fluid (CSF) containing immune cells that differ markedly from the peripheral blood,^8^ the anterior chamber of the eye is bathed in aqueous fluid that is normally devoid of immune cells. During uveitis, the blood-ocular barrier breaks down, immune cells traffic into the eye and the intraocular immune response becomes clinically evident using an ophthalmic microscope called a slit lamp.^9^^,^^10^^,^^11^ While the existence of a triggering antigen has been postulated for more than a century^12^ and modeled in mice,^13^ little direct evidence exists to implicate an antigenic trigger in human uveitis.

While the small volume of aqueous fluid in the anterior chamber of the eye (100–200 μL) previously hampered the study of human ocular immune cells, recent transcriptomic advances have made in-depth analysis of small volume samples possible. Three groups have thus far utilized scRNA-seq to study the immune cells present in ocular fluid biopsies from small cohorts of patients with discrete clinical disease phenotypes.^14^^,^^15^^,^^16^ Each study identified key inflammatory cells specific to a small subset of uveitis, and collectively, these studies suggest that the composition of inflammatory cells may vary between clinical phenotypes. We hypothesized that direct comparison of multiple uveitis subtypes would allow comprehensive mapping of the landscape of ocular immune response in human uveitis and identification of immunologic features unique to each subtype. To this end, we used transcriptomic and immune receptor repertoire analysis to profile the ocular and peripheral blood immune cells from 23 patients with diverse clinical uveitis phenotypes.

## Results

### Clinical features and demographics

We obtained ocular fluid samples from 23 patients, with active intraocular inflammation, including cellular infiltrate in the anterior chamber, two of whom were sampled twice during separate disease flares. Clinical and sample data are provided in Table S1. The average age was 46 years; 70% were female; and 52% were black Americans. The most common diagnosis was HLA-B27 AAU, present in 17%, while 56% of cases remained undifferentiated after a standard laboratory evaluation (see STAR Methods). Among patients with HLA-B27 AAU, two patients (UV027 and UV122) also had axial spondyloarthritis and one case (UV180) had a dense collection of inflammatory cells, or hypopyon, in the anterior chamber. Most patients had inflammation restricted to the anterior portion of the eye (anterior and intermediate uveitis); however, four patients had active posterior inflammation that included either retinal vasculitis (UV284) or choroiditis (UV176, UV186, UV195). Forty-eight percent of cases followed a chronic disease course and 68% of patients were using topical steroids at the time of sampling, at an average of 2 drops prednisolone per day. Thirty-two percent were using systemic anti-inflammatory therapy at the time of sampling and 74% ultimately required long-term systemic immunosuppression to control their disease.

### The landscape of ocular immune cells in uveitis

**Figure 1:**
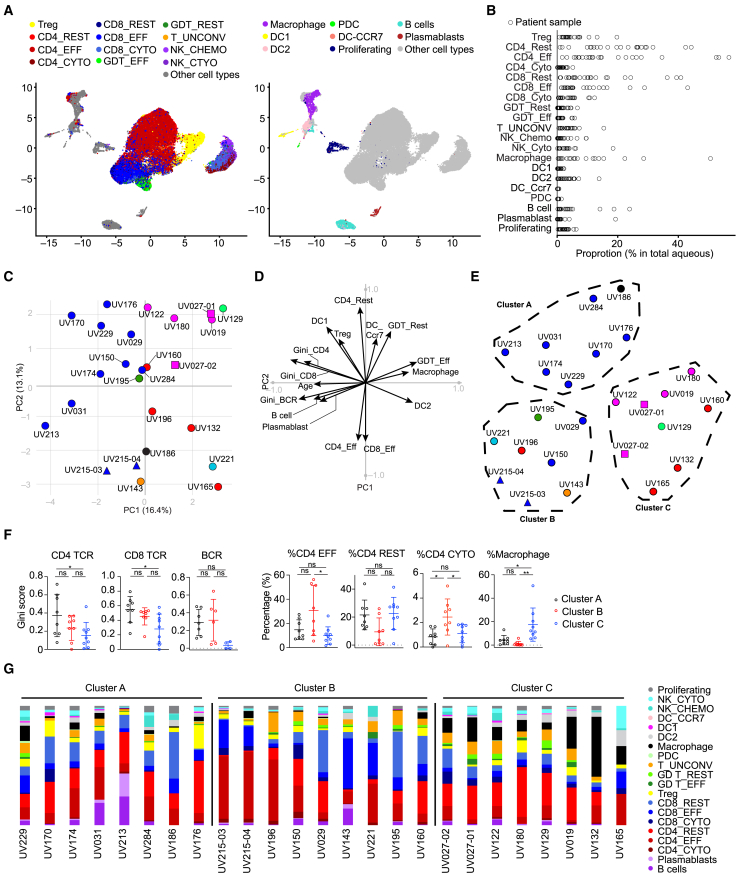
The composition of ocular immune cell types varies between clinical disease phenotypes (A) Uniform Manifold Approximation and Projection (UMAP) representation of ocular immune cells detected in ocular biopsies. Left: T cell and NK cell clusters; Right: myeloid and B cell and proliferation clusters. x axis UMAP 1; y axis UMAP 2. (B) Distribution of the proportions of each cell type across samples. (C) Principal component (PC) 1 vs. PC2 from principal component analysis (PCA) of cell type proportions, Gini coefficients and clinical features for 25 samples showing separation of samples by clinical diagnosis. Diagnoses are depicted by color (blue: undifferentiated uveitis with granulomatous KP, dark green: acute Vogt-Koyanagi-Harada’s (VKH) with granulomatous KP, light green: chronic VKH without granulomatous KP, black: birdshot chorioretinitis, orange: juvenile idiopathic arthritis, red: undifferentiated uveitis without granulomatous KP, teal herpetic uveitis, pink: HLA-B27 acute anterior uveitis (AAU). Repeat samples from the same patient are depicted by squares (UV027-01 and 02) and triangles (UV215-03 and 04). (D) Vector plot showing the magnitude and directionality of each variable on the distribution of patient samples in the space defined by PC1 and PC2 showing adaptive and innate immune features drive sample differences in PCA. (E) UMAP showing distribution pattern of patient samples based on the first 10 PCs. Diseases are depicted by color denoted in C. Repeat samples from the same patient are depicted by squares and triangles as in C. x axis UMAP 1; y axis UMAP 2. (F) Distribution of Gini scores and proportions of cell types across the three patient clusters as defined in (E) represented as mean ± SEM. Ordinary one-way ANOVA with Tukey multiple comparison test was used for statistical analysis, with *p* value >0.05 denoted as ns, *p* value ≤0.05 as ∗, *p* value ≤0.01 as ∗∗, and *p* value ≤0.001 as ∗∗∗, and *p* value ≤0.0001 as ∗∗∗∗. (G) Proportions of immune cell types for each sample arranged by cluster, showing Clusters A and B are enriched in lymphocytes, while cluster C is enriched in myeloid cells. See also, Figure S1 and Tables S1 and S2.

We performed paired single-cell RNA-seq (scRNA-seq) and V(D)J sequencing on 25 ocular fluid samples from 23 patients with uveitis. In total, we obtained 44,007 scRNA-seq profiles after quality control filtering. Dimensionality reduction yielded 20 cell state clusters, which we annotated based on expression of cluster-defining and canonical genes. Most cells belonged to one of seven main cell types: CD4 T cells, CD8 T cells, unconventional T cells (including γδ T cells and mucosal-associated invariant T cells (MAIT), NK cells, dendritic cells (DCs), macrophages, and B cells (Figures 1A, S1A, and S1B).

Consistent with prior flow cytometry^9^ and smaller-scale scRNA-Seq analyses,^14^^,^^15^^,^^16^ we found that CD4 T cells were the predominant leukocyte in most ocular infiltrates (Figure 1A). We were able to distinguish resting (*TCF7*, *CCR7*) and effector (*CXCR6*, *TBX21*, and *RORC*) CD4 T cells, including a subset of effector CD4 T cells with cytotoxicity genes (*PRF1* and *GNLY)* (Figures 1A, S1A, and S1B). Regulatory T cells (*FOXP3* and *IL2RA*) were also detected. Among CD8 T cells, we identified a resting cluster (*CD27* and *TCF7)*, an effector cluster (*CCL4* and *GZMK)*, and a small cytotoxic cluster (*PRF1* and *GNLY)*. We also found several types of unconventional T cells, including both resting (*LEF1*, *TCF7*, and *XCL1*) and effector (*PRF1* and *GNLY*) γδ T cells, which have been indicated in the pathogenesis of both human uveitis and animal models,^17^^,^^18^^,^^19^ as well as a mixed cluster of other unconventional T cells (including MAIT cells expressing *KLRB1*, *ZBTB16*, and *TRAV1-2*). We identified 2 populations of NK cells, one with prominent cytotoxicity genes (*PRF1*, *GNLY*, and *GZMB*), and the other highly expressing chemokine genes (*CCL3*, *CLL4*, and *CCL5*). Macrophages (*CD14*, *VCAN*, and *S100A8*) were the most abundant myeloid cells (Figures 1A, S1A, and S1B). We also identified type 1 (*XCR1* and *CLEC9A*) and type 2 (*CD1C* and *CLEC10A*) conventional DCs, as well as plasmacytoid DCs (*SERPINF1* and *GZMB*) and a small population of CCR7+ DCs *(CCR7*, *LAMP3*, and *CCL19)* (Figures 1A, S1A, and S1B). Finally, we identified B cells (*CD79A*, *CD19*, and *MS4A1*) and plasmablasts (*CD79A*, *MZB1*, and *XBP1*) (Figures 1A, S1A, and S1B).

### The composition of immune cell types varies between clinical disease phenotypes

The frequency of each ocular immune cell type varied greatly between samples (Figure 1B; Table S2). Overall, the most abundant cell type, the fraction of T cells in individual samples varied widely across our cohort from 35% to 88% of the total infiltrate (Figure 1B; Table S2). Many patients with undifferentiated uveitis, and in particular those with dense cellular aggregates on the inner corneal surface referred to as granulomatous-appearing keratic precipitates (KP), had very high proportions of CD4 T cells (50–64%, Table S2). Patients with HLA-B27 AAU had some of the lowest CD8 fractions (7–15%, Table S2). Myeloid cells, predominantly macrophages, also varied widely from less than 1% to more than 50% of each ocular infiltrate and were notably abundant in several patients with HLA-B27 AAU (22–33%, Table S2), consistent with prominence of myeloid cells in HLA-B27 AAU found by Kasper et al.^14^ B cells were undetectable in some patients but comprised up to 43% in some patients with undifferentiated uveitis with granulomatous KP. We also analyzed the extent of T and B cell expansion in each sample by quantifying both the fraction of total T or B cells that were expanded and a Gini coefficient or measure of inequality in frequencies within a sample (Figure S1G). This variability of immune cell types between samples from patients with discrete clinical phenotypes suggested that clinically defined disease subtypes might differ in the composition of their ocular inflammatory infiltrates.

To address this, we utilized principal-component analysis (PCA) to study 26 variables including the proportion of each immune cell type, the clonality (extent of clonal expansion) of CD4 and CD8 T cells and B cells, and clinical parameters that might influence ocular immune responses, specifically patient age, duration and severity of disease, and use of local steroid treatment. The first 10 PCs accounted for 93% of the variance between samples (Figure S1C) and the separation of patient samples across the first 2 PCs revealed several clinically meaningful trends. First, samples from the same eye taken at different disease flares clustered together (Figure 1C UV027-01 and -02 and UV215-03 and -04), suggesting that immunologic features may be fairly stable between disease flares. In contrast, unique patient samples were distributed such that PC1 largely separated cases of undifferentiated uveitis with granulomatous KP from other cases (Figure 1C). Most notably, samples from patients with HLA-B27 AAU, separated from most other cases across PC1 and PC2 (Figure 1C). This indicated that a combination of immunologic features distinguished clinical disease subtypes in our cohort.

We then asked which features drove this distinction by plotting each variable as a vector in the PC1 by PC2 space such that the magnitude and direction indicate the influence of each variable on the distribution of patient samples (Figure 1D, for contribution of all variables to each dimension, see Figure S1D). While patient age was a significant variable in our analysis, duration, severity, and steroid treatment were not (Figure S1E).

Adaptive immune features, including higher proportions and increased clonality of CD4 T cells, CD8 T cells and B cells most strongly drove the separation across PC1 of undifferentiated uveitis with granulomatous KP from HLA-B27 AAU and other cases lacking granulomatous KP, which were more strongly associated with innate features including macrophages, DCs and unconventional T cells (Figure 1D). This distinction is consistent with prior studies identifying antigen-driven immune responses in a specific granulomatous uveitis subtype, Vogt-Koyanagi-Harada disease (VKH)^20^ as well as the prominence of myeloid cells in HLA-B27 AAU found by Kasper et al.^14^ and in synovial infiltrates from another HLA-B27-associated disease, ankylosing spondylitis.^21^^,^^22^^,^^23^

Next, we applied the first 10 PCs to Uniform Manifold Approximation and Projection (UMAP) analysis^24^ and defined three clusters based on the distribution of the samples (Figure 1E). Cluster A demonstrated the greatest patient age, as well as a relatively high CD4, CD8, and B cell clonality and higher percentage of DC1 (Figures 1F–1G and S1F). The ocular infiltrates in cluster B had intermediate levels of T cell expansion, but high B cell expansion, the highest percentages of effector CD4 and CD8 T cells and the lowest frequency of myeloid cell types (Figures 1F–1G and S1F). Cluster C samples had the lowest T and B cell clonality, but the highest percentage of macrophages and DC2, and were relatively enriched, along with cluster B in unconventional T cells (Figures 1F–1G and S1F). Thus, our three clusters recapitulated the most significant variables in PC1 and PC2, where Cluster A most strongly demonstrated multiple features of adaptive immunity; Cluster B was particularly enriched in effector T cells; and Cluster C was distinguished by a predominance of innate immune cell types.

The clusters also distinguished disease subtypes (Figure 1E). All HLA-B27 AAU samples grouped together in cluster C, with most cases of undifferentiated uveitis presenting without granulomatous KP. Most undifferentiated cases with granulomatous KP fell into cluster A. Cluster B also included samples from patients with undifferentiated uveitis with granulomatous KP including VKH as well as another chronic disease with adaptive immune dysregulation, juvenile idiopathic arthritis-associated uveitis.^20^^,^^25^^,^^26^^,^^27^

In summary, deep transcriptional profiling of ocular immune cells has resolved immunologic distinctions between clinical uveitis entities. Our data suggest that adaptive immune cell types are more prominent in ocular infiltrates from patients with granulomatous clinical features, while innate immune cells are more prominent components of the ocular infiltrate in non-granulomatous types of uveitis.

### Ocular T cells are clonally expanded by local antigen stimulation

**Figure 2:**
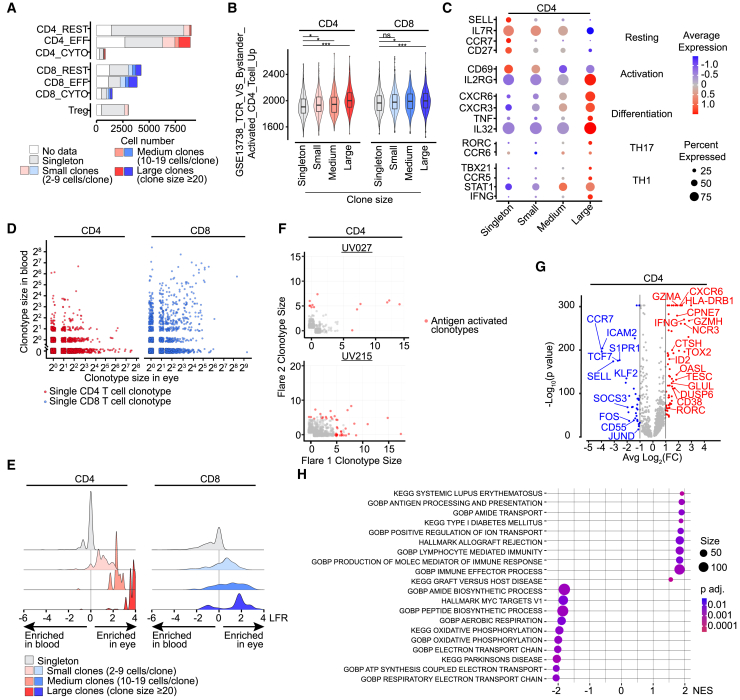
Ocular T cells are clonally expanded by local antigen stimulation (A) Clonal expansion within ocular CD4 and CD8 cell types, as shown by absolute numbers contributed by singlets (clone size = 1, gray), small clones (2–9 cells/clone, light red/blue), medium clones (10–19/clone, medium red/blue) and large clones (clone size ≥20, dark red/blue). Cells with transcriptional data but no TCR data were marked as “no data” (white). (B) Single cell gene set enrichment (GSEA) of T cell receptor signaling genes GSEA13718 in ocular CD4 (left) and CD8 (right) T cells, by clonotype size group (singleton, small, medium, and large, as defined in A). One-way ANOVA was used for statistical analysis, with *p* value >0.05 denoted as ns, *p* value ≤0.05 as ∗, *p* value ≤0.01 as ∗∗, and *p* value ≤0.001 as ∗∗∗, and *p* value ≤0.0001 as ∗∗∗∗. (C) Expression of activation/differentiation genes by ocular CD4 T cells. Clonotype size groups: singleton, small, medium, and large, as defined in A, (color indicates relative level and circle size indicates percent express). (D) Frequency in eye (x axis) vs. blood (y axis) of each CD4 (left) and CD8 (right) clonotype (each dot represents a unique clonotype). (E) Distribution of enrichment scores (log-fold ratio (LFR) of the frequency of cells in each clonotype in the eye compared to blood) for CD4 (left) and CD8 (right) T cells clonotypes by size groups (from top to bottom: singleton, small, medium, and large, colors in key and correspond to A and B). (F) Antigen-activated CD4 clonotypes were present in both flares. Clonotype frequency in flare 1 (x axis) vs. flare 2 (y axis) for each CD4 clonotype in two patients (eyes) that underwent serial sampling. Red indicates antigen-activated clonotypes (size >/ = 5 cells; LFR>1 in at least one flare) and gray indicates clonotypes that are not enriched in either flare. Patient UV027 (HLA-B27 AAU, top) and UV215 (undifferentiated uveitis with granulomatous KP, bottom). (G) Differential gene expression between all antigen-activated CD4 T cells (size >/ = 5 and LFR>1) and singleton CD4 T cells. (H) Gene set enrichment analysis (GSEA) of differentially expressed genes (panel G) between antigen-activated CD4 T cells and singleton CD4 T cells. NES normalized enrichment score, high scores are associated with antigen-activated CD4 T cells. See also Figure S2.

Given the prominence of adaptive immune features in a subset of our cohort, we sought to characterize the ocular immune response to antigen in human uveitis. Each ocular sample (100–200 μL) represented 30–50% of the total aqueous fluid volume in the anterior chamber. T cells represented a variable fraction of the total ocular infiltrate (Figure 1). The size of individual T cell clonotypes, or groups of clonally expanded T cells with identical T cell receptors, TCRs, varied from 1 cell to more than 100 cells, with the largest clonotypes representing more than 10% of their respective total ocular T cell populations. (Figures 2A and S2A). We defined large clonotypes as those containing 20 or more cells, medium clonotypes of 10–19 cells, and small clonotypes as containing 2–9 cells.^28^ Within the eye, effector CD4 T cells contained the greatest number of clonally expanded T cells, followed by effector and resting CD8 T cells (Figure 2A). In contrast to ocular T cells, clonal expansion in peripheral blood samples was rare except in memory CD8 T cells (Figure S2B), which may represent large oligoclonal expansion of antiviral CD8 effector memory cells in healthy individuals.^29^^,^^30^

Next, we asked whether the presence of large clonotypes indicated local antigen-driven clonal expansion by assessing the expression of TCR signaling-induced genes.^31^ Among CD4 T cells, increasing clonotype size was associated with expression of TCR signaling genes (Figure 2B), suggesting that large ocular CD4 clonotypes may have expanded locally in response to ocular antigen. Increased clonotype size was also associated with features of activation and Th1/17 differentiation (Figure 2C). Singleton CD4 T cells preferentially expressed resting features (SELL, IL7R), while medium and large clonotypes preferentially expressed genes associated with activation (IL2RG),^32^ effector cytokines (TNF, IL32), Th1 differentiation (TBX21, IFNG),^33^^,^^34^ and Th17 differentiation (RORC, CCR6).^33^ Large clones also expressed high CCR6, a surface marker most often seen in Th17 cells, especially in an autoimmune setting,^35^^,^^36^ as well as CCR5, which is expressed most abundantly on Th1 cells.^37^^,^^38^

Among CD8 T cells, clonotype size minimally correlated with expression of TCR activation genes (Figure 2B), but was associated with gene expression suggesting a history of activation (Figure S2C). The weaker association between clonotype size and TCR signaling in CD8 T cells raised the possibility that the ocular CD8 population may be heterogeneous, containing a mixture of immigrants of previously expanded peripheral blood cells as well as intraocularly expanded clonotypes.

To investigate this possibility, we compared the frequency of T cells within each clonotype between the eye and blood. Among CD4 T cells, clonotype size and ocular enrichment were closely correlated, with the largest ocular clonotypes also highly enriched in the eye (Figures 2D and 2E), supporting local antigen-driven expansion. Among CD8 T cells, the correlation was weaker. A subset of large CD8 clonotypes appeared enriched in the eye (Figures 2D and 2E) suggesting they too may expand in response to local antigen. However, a significant fraction of large ocular CD8 T cell clonotypes were highly abundant in the corresponding peripheral blood sample (Figures 2D and 2E), suggesting they migrated into the eye as previously expanded blood clonotypes.

Thus, ocular CD4 and CD8 T cells demonstrate differing patterns of clonal expansion, enrichment and expression of genes associated with TCR signaling. Among CD4 T cells, there was strong correlation between clonotype size, ocular enrichment and TCR gene expression, indicative of a local antigen-driven immune response. While a subset of CD8 T cells were also likely activated by local antigen, clonotype size was poorly correlated with ocular enrichment and expression of TCR activation genes.

Given this discrepancy, we asked whether ocular enrichment was a better proxy for TCR-stimulation than clonotype size. To explore this, we assessed the expression of TCR signaling genes in clonotypes with increasing enrichment in the eye vs. blood (Figure S2D) and found that enrichment in the eye was associated with TCR stimulation genes in both CD4 and CD8 clonotypes (Figure S2E). This indicates that both clonotype size and tissue enrichment are important considerations for identifying CD8 T cells likely to have undergone local antigen-stimulation. In fact, for CD8 T cells, enrichment was superior to clonotype size at discriminating T cells with gene expression indicative of recent TCR stimulation.

With this framework in mind, we set a threshold for identifying the ocular T cells mostly likely to have been expanded by local antigen as ocular clonotypes comprised of at least 5 cells, which were also enriched in the eye by log-fold enrichment (LFR) > 1 compared to the peripheral blood. The fraction of antigen-activated T cells correlated strongly with the extent of clonal expansion among CD4 T cells as assessed by Gini coefficient in each sample (Figure S2F) but not with the total proportion of T cells (Figure S2G), supporting the notion that a subset of ocular T cells are recruited and/or activated by non-antigenic triggers. Furthermore, identical CD4 T clonotypes were present in serial disease flares (Figure 2F), suggesting that antigen-specific CD4 T cells remain in the eye or return to the eye between flares. We also detected antigen-activated CD8 clonotypes in subsequent uveitis flares from one patient (UV215), although most expanded CD8 T cells were unique to separate disease flares (UV027) or related to highly expanded blood clonotypes (UV215) (Figure S2H). Together, these data suggests that ocular antigens can drive local clonal expansion and activation of T cells in uveitis.

We next asked which genes and biological processes were unique to the cells that had likely undergone antigen-driven clonal expansion in the eye. Compared to non-expanded singleton T cells, antigen-activated CD4 T cells demonstrated higher expression of genes including *CD38*, *CTSH*,^39^ *GLUL*,^40^ *CXCR6*,^36^ *IFNG*, and *RORC* (Figure 2G), suggesting that ocular antigen stimulation drives a Th1/17 effector differentiation. The transcriptional program in antigen-activated ocular CD4 T cell also overlapped with other antigen-driven inflammatory diseases including allograft rejection, type 1 diabetes, graft vs. host disease and systemic lupus erythematosus (Figure 2H), suggesting that ocular CD4 T cells share pathologic features with CD4 T cells in other autoimmune diseases. While the differential gene expression in antigen-activated CD8 T cells was less robust than for CD4 T cells, increased expression of *CD38*, *GLUL*, *CXCR6*, and *IFNG* by these cells (Figure S2I) supports to the notion that a subset of ocular CD8 T cells have also undergone TCR-simulation^41^ as suggested by our previous work.^42^

In summary, we found that clonally expanded and eye-enriched T cells display evidence for local antigen-stimulated activation and differentiation. Specifically, the transcriptional program of expanded ocular CD4 T cells indicates local antigen-induced expansion and differentiation into Th1/17 effector cells. Likewise, a subset of highly expanded ocular CD8 T cells likely also responded to local antigen while other large ocular CD8 clonotypes likely immigrated to the eye as pre-expanded peripheral blood CD8 T cells.

### Antigen-activated T cells associate with key clinical features

**Figure 3:**
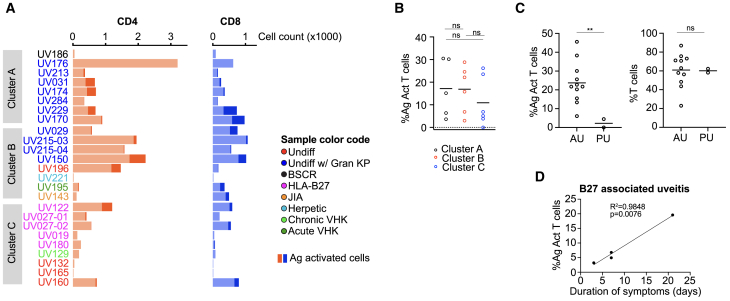
Antigen-activated T cells associate with key clinical features (A) Quantification of antigen-activated CD4 (left, orange) and CD8 (right, blue) T cells as a percentage of total T cells in each aqueous sample. Color of patient labels indicates disease type (see figure key). (B) Percentage of antigen activated T cells (CD4 and CD8 T cells combined) in each cluster defined in Figure 1. The mean in each cluster is marked. Samples with fewer than 50 antigen-activated CD4 or CD8 T cells were excluded. Ordinary one-way ANOVA with Tukey multiple comparison test was used for statistical analysis, with *p* value >0.05 denoted as ns, *p* value ≤0.05 as ∗, *p* value ≤0.01 as ∗∗, and *p* value ≤0.001 as ∗∗∗, and *p* value ≤0.0001 as ∗∗∗∗. (C) Increased proportion of antigen activated T cells of all T cells (left) in anterior uveitis samples (AU) vs. panuveitis samples with more prominent posterior inflammation (PU), but no difference in proportion of all T cells (right) between AU and PU. Samples with fewer than 50 antigen-activated CD4 or CD8 T cells were excluded. AU included: UV229, UV215-04, UV215-03, UV213, UV196, UV174, UV170, UV150, UV143, UV031, and UV029. PU group included: UV176 and UV284. Welch’s t test was used for statistical analysis, with *p* value >0.05 denoted as ns, *p* value ≤0.05 as ∗, *p* value ≤0.01 as ∗∗, and *p* value ≤0.001 as ∗∗∗, and *p* value ≤0.0001 as ∗∗∗∗. (D) Increase in proportion of antigen activated T cells with increasing duration of inflammation (in days) among 5 samples from patients with HLA-B27 AAU. Regression analysis was done using simple linear regression. See also, Figure S3.

The proportion of antigen-expanded T cells in each infiltrate varied across patients in our cohort from 0% to 30%, with higher proportions in patients from clusters A and B (Figures 3A, 3B, and S3E), corresponding to the increased prevalence of adaptive immune features we identified in Figure 1. Several additional clinical features may drive immunologic differences between patients in our cohort.

First, the frequency of antigen-activated aqueous T cells was greater in samples from patients with anterior uveitis in which inflammation is concentrated in the anterior chamber of the eye (denoted as “AU” in Figures 3D and S3C) compared to those from patients with panuveitis with more prominent posterior inflammation (denoted as “PU” in Figures 3D and S3C). Given that our samples were obtained from the anterior chamber of the eye, this finding suggests that antigen-activated T cells may localize near the primary site of clinical inflammation (i.e., the anterior chamber in anterior uveitis), and that in more distal compartments of the eye (i.e., the anterior chamber in posterior/pan uveitis) T cells may be non-specifically activated.

Second, the frequency of antigen-activated T cells may be increased with a longer duration of ocular inflammation. Among patients with HLA-B27 AAU, the frequency of antigen-activated T cells also increased with disease duration (Figures 3E and S3D), ranging from 0 to 7% in most samples, but was 20% in one patient with chronic, poorly controlled uveitis. Furthermore, antigen-activated T cells were also abundant in a patient with chronic uveitis associated with juvenile idiopathic arthritis (JIA) and in several cases of chronic uveitis with granulomatous KP (Figure 3A). Thus, adaptive immune responses in uveitis, and specifically antigen-activated T cells, may be compartmentalized within the eye and become amplified during chronic inflammation.

### Innate activation of myeloid cell types distinguishes HLA-B27 AAU

**Figure 4:**
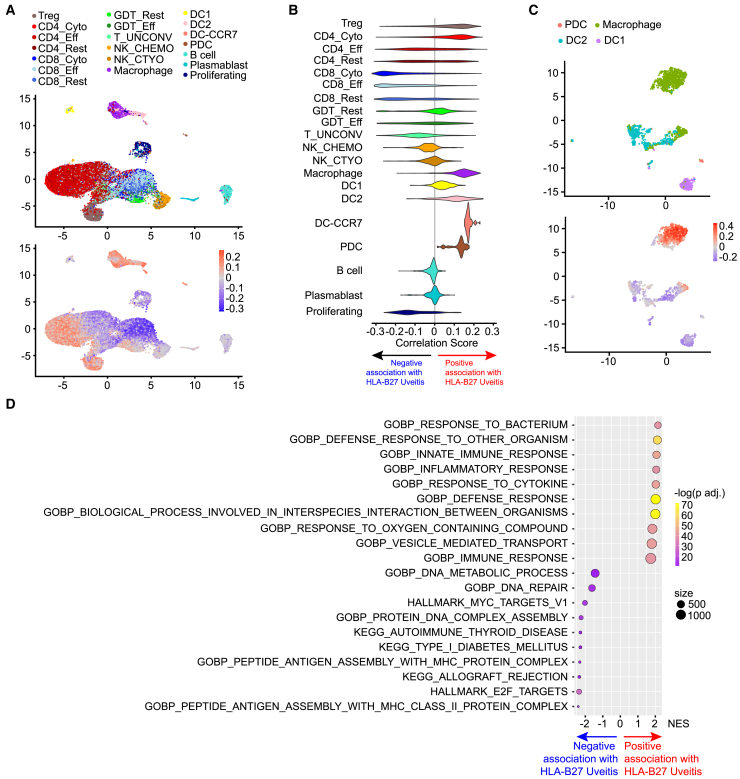
HLA-B27 associated acute anterior uveitis (AAU) features innate activated myeloid cells (A) Association between ocular immune cell states and HLA-B27 AAU determined by covarying neighborhood analysis (CNA): UMAP cluster identities (top) and UMAP association testing results for HLA-B27 across all cells (bottom), colored according to neighborhood coefficient (red: high correlation, blue: low correlation). (B) Distribution of HLA-B27 neighborhood coefficient values within each original cluster. (C) Association between ocular myeloid cell states and HLA-B27 AAU determined by CNA: UMAP cluster identities (top) and association for HLA-B27 across all myeloid cells (bottom), colored according to neighborhood coefficient (red: high correlation, blue: low correlation). (D) GSEA analysis of the genes driving myeloid cell CNA in C.

HLA-B27 AAU has a robust clinical phenotype with rapid onset of severe inflammation and strong genetic factors including HLA-B27 and ERAP1.^43^ Not surprisingly, samples from patients with HLA-B27 AAU clustered together in our data, suggesting that they share key immunologic features in addition to their clinical phenotype. To identify the immune cell states specifically correlated with HLA-B27 AAU, we used covarying neighborhood analysis (CNA). CNA identifies small neighborhoods of transcriptionally related cells that co-vary in abundance across samples.^44^ Because the associations are based on highly granular cell groups, CNA is more sensitive and nuanced for identification of variance across samples than Seurat-based analyses of cell type abundance. To avoid sample bias, we removed repeat samples from the same patients (UV027 and UV215). Consistent with our PCA and clustering analyses, we found the highest correlation with HLA-B27 AAU in cell neighborhoods contained within myeloid cells (Figures 4A and 4B) as well as regulatory T cells and cytotoxic CD4 T cells. In contrast, CD8 T cell clusters had low correlation scores, indicating that these cell states were of relatively lower abundance in HLA-B27 AAU samples compared to other samples in our cohort (Figures 4A and 4B).

We next asked whether specific myeloid cell states were associated with HLA-B27 AAU. To obtain a more granular analysis, we performed CNA on isolated myeloid cells (LYZ+) and found cell neighborhoods in the largest macrophage cluster to be most associated with HLA-B27 AAU, while neighborhoods in the DC1 cluster were least associated with HLA-B27 AAU (Figure 4C). Gene set enrichment analysis (GSEA) revealed high expression of genes associated with innate immunity in HLA-B27 ocular myeloid cells, and lower expression of genes associated with antigen presentation and adaptive immunity (Figure 4D). Furthermore, we found T cells from ocular infiltrates in HLA-B27 AAU were relatively depleted of genes associated with adaptive immune responses compared to other uveitis samples (Figures S4A and S4B). Altogether, our data indicate a prominent innate immune response among aqueous immune cells during HLA-B27 AAU. This may, in part, be associated with the acuity of this disease as most samples in our cohort were obtained early in the disease flare, possibly before extensive expansion of antigen-reactive T cells such as those identified by Yang et al.^42^

## Discussion

Uveitis is a heterogeneous group of diseases in which clinical phenotypes vary significantly. The current study sheds light on the immunologic mechanisms that may contribute to this diversity. Prior immunophenotyping studies of small uveitis cohorts^14^^,^^15^^,^^16^ suggested that the composition of ocular immune cells might correlate with clinical phenotypes, yet a direct comparison of multiple disease types had not been undertaken. Here, we analyzed the ocular inflammatory infiltrates from 23 patients with various clinical phenotypes using scRNA-seq paired with immune repertoire profiling. The composition of the ocular inflammatory infiltrates, specifically the prevalence and/or clonal expansion of key cell types, drove clustering of patient groups with distinct clinical phenotypes and revealed within-group heterogeneity. This study reveals that distinct immunologic mechanisms drive disease in discrete uveitis subtypes, and suggests that targeted therapies based on immunologic classification, rather than clinical features, may be beneficial.

### The role of ocular antigen in uveitis

In 1910, Anton Elschnig, drew upon observations that trauma in one eye could trigger granulomatous inflammation in the fellow “sympathizing” eye to design a series of experiments demonstrating the existence of an ocular antigen.^12^ Despite significant efforts over the intervening century, however, the identity of the antigen(s) and their specific role(s) in human ocular inflammation remain unknown. Using both single cell transcriptional and immune receptor profiling, and including matched peripheral blood samples as control, our study is the first of its kind to provide evidence for local antigen-driven expansion of T cells in uveitis. We identified antigen-activated ocular T cells as (1) clonally expanded, (2) enriched in the eye compared to the peripheral blood, and (3) expressing genes downstream of TCR ligation. Furthermore, we found these antigen-activated T cells in cases of granulomatous uveitis, even in the absence of prior ocular trauma, suggesting that the presence of intraocular granuloma-like lesions may be a clinical sign that indicates antigen-driven inflammation.

Our findings support and expand upon those of Kang et al.^16^ describing clonal expansion of CD4 T cells in 4 patients with Vogt-Koyanagi-Harada’s disease (VKH), a granulomatous uveitis characterized by an immune response targeting melanocytes.^20^ Using a stringent definition including expansion, local enrichment and expression of TCR signaling genes, we identified antigen-activated T cells in a patient with acute VKH as well as in other patients with granulomatous uveitis. Furthermore, we identified additional pathologic correlates of granulomatous inflammation, including clonal expansion of ocular B cells that support a strong role for antigen.

Our analysis revealed additional heterogeneity within uveitis subtypes. We found increased antigen-activation when the clinically apparent inflammation was proximal to the sampled site (i.e., the anterior chamber) rather than distal (i.e., the retina or choroid). We also found increased antigen-activation associated with disease chronicity. Together, this suggests that within the eye, antigen-driven inflammation may vary across ocular compartments and evolve over time.

### Unique roles for ocular CD8 T cells

A subpopulation of clonally expanded ocular CD8 T cells showed ocular enrichment along with TCR gene expression, indicative of local antigen-driven expansion. In contrast, many clonotypes were expanded *a priori* in the peripheral blood. For the latter group, several possibilities exist regarding their mode of activation. Pre-expanded clonotypes may have been activated by cognate antigen in the periphery and contributed to ocular inflammation in a bystander manner, independent of cognate antigen stimulation. Alternatively, clonotypes initially activated in the periphery, perhaps by microbiota as suggested by Yang et al.^42^ and some uveitis models,^45^^,^^46^ may have been sampled before intraocular expansion could occur or may be subject to intraocular immunoregulation preventing significant expansion.

One intriguing observation involves the granzyme expression patterns of the CD8 T cells. While ocular CD8 T cells expressed a low level of the apoptosis-inducing granzyme, GZMB, they expressed high levels of inflammation-promoting GZMA and GZMK.^47^ A similar GZMB^lo^GZMK^hi^ CD8 population was identified in the synovial fluid in rheumatoid arthritis, having lower cytotoxic potential than the GZMB^hi^ CD8 T cells in the blood, and was able to drive reactive oxygen species (ROS) and inflammatory cytokine production of fibroblasts *in vitro.*^48^ This non-canonical cytotoxicity profile suggests that instead of inducing apoptosis, ocular CD8 T cells might play a role in initiating or sustaining the inflammatory tone in uveitis.

### HLA-B27 associated acute anterior uveitis features innate activated myeloid cells

HLA-B27 AAU is a distinct clinical entity featuring acute and highly symptomatic episodes of inflammation that alternate between eyes. In agreement with this clinical distinctness, we found discrete cell states that were highly correlated with HLA-B27 AAU, most significantly macrophages and other myeloid cell types. Kasper et al.^14^ also identified myeloid subtypes in samples from both HLA-B27-positive and -negative anterior uveitis as well as bacterial endophthalmitis. In agreement with the acute nature of the disease, HLA-B27-associated ocular myeloid cells were enriched in innate immune response genes and relatively depleted in antigen presentation and T cell activation genes. Accordingly, most samples from patients with HLA-B27 AAU had relatively small fractions of antigen-activated T cells and these clonotypes varied between serial samples from the same patient, suggesting some degree of stochasticity to the inflammation. Our results suggest that, for HLA-B27 AAU, myeloid cells play a key role early in the intraocular inflammatory response, in line with other studies of HLA-B27-associated disease.^49^

Similar to HLA-B27 AAU in our study, Kang et al.^16^ identified a relative myeloid predominance in ocular infiltrates from patients with Bechet’s disease, an HLA-B51-associated autoinflammatory disease that also features acute severe anterior chamber inflammation.^50^^,^^51^ They also found large predominantly CD8 ocular clonotypes, although they did not assess their enrichment relative to the blood. Nevertheless, the coexistence of myeloid-rich infiltrates and antigen-activated T cells in both HLA-B27 AAU and Behcet’s disease supports their designation by the EULAR study group as MHC-opathies, diseases in which polymorphisms in antigen presentation genes are hypothesized to predispose patients to tissue inflammation driven by presentation self-peptides by myeloid cells to CD8 T cells.^52^

The extent to which our observed differences represent disease-specific immunologic mechanisms remains to be determined by future analyses using a larger dataset. Collectively, these studies indicate that individual cases may fall along a spectrum of autoinflammation to antigen-driven autoimmunity. Deep immune profiling offers new insights into the types of immune responses driving uveitis in individual patients and may eventually identify precision therapeutic strategies.

### Limitations of the study

We acknowledge several limitations of this study. While this is the largest cohort of uveitis patients studied using single cell RNA sequencing to date, it is still a relatively small group considering the heterogeneity which exists both between defined clinical entities and between patients with the same disease. Adding to this complexity, additional factors such as age, genetic and environmental risk factors, and prior treatment, likely influence the immune responses. Larger cohort sizes may increase the variance in future PCA and result in tighter clustering in UMAP analyses. Because limited cell numbers in some samples might have limited the robustness of certain analysis, we excluded samples with low cell numbers in our analyses of clonal expansion and antigen-activated T cells. Despite the limitations, our data revealed patterns that were reproducible within our dataset and well correlated with prior studies. In future studies, protein-level expression or spatial information might provide increased insight into the interaction between different immune cell types and tissue. Our analysis was limited to the fluid biopsied from the anterior chamber of the eye and may imperfectly reflect pathology in ocular tissues and distal regions of the eye, such as the vitreous, retina or choroid. However, considering the visual morbidity associated with solid tissue biopsy, small volume ocular fluid biopsy from the anterior chamber of the eye is currently the most reasonable way to obtain samples for the study of uveitis. We predict that as the number of samples analyzed by this and other advanced molecular profiling techniques grow, pathophysiologic insights that lead to precision therapies will become apparent.

## Resource availability

### Lead contact

Further information and requests for reagents should be directed to and would be fulfilled by the lead contact, Lynn M. Hassman (lynn.hassman@cuanschutz.edu).

### Materials availability

This study did not generate new unique reagents.

### Data and code availability

• RNA-sequencing data from human aqueous and peripheral blood cells are deposited in the Gene Expression Omnibus (GEO) database and are publicly available. Accession numbers are listed in the key resources table.
• This paper does not report original code.
• Any additional information required to reanalyze the data reported in this paper is available from the lead contact upon request.

## Acknowledgments

L.M.H. was supported by 10.13039/100000002NIH: K08EY033045 and P30-AR073752; L.M.H., C.C., Y.X., A.T., Y.K., G.W.B., and P.A.R. were supported by 10.13039/100001818Research to Prevent Blindness; M.A.P. was supported by 10.13039/100000002NIH, K08AR079593 and P30-AR073752; the 10.13039/100000964Arthritis National Research Foundation. In addition to the listed authors, Dr. Wayne Yokoyama provided mentorship and technical support for sample collection.

## Author contributions

C.C.: methodology, software, validation, formal analysis, data curation, visualization; Y.X.: formal analysis, visualization, writing—original draft, review and editing; Y.K.: investigation; G.W.B.: investigation; A.T.: investigation; M.A.P.: resources, writing—review and editing; P.A.R.: writing—review and editing; L.M.H.: conceptualization, methodology, investigation, formal analysis, resources, writing—original draft, review and editing, supervision.

## Declaration of interests

The authors declare no conflicts of interest.

## STAR★Methods

### Key resources table

**Table:** 

REAGENT or RESOURCE	SOURCE	IDENTIFIER
**Deposited data**		

scRNA-Seq data	GEO	GSE229166
scVDJ-Seq data	GEO	GSE274471

**Software and algorithms**		

10x Genomics Cell Ranger v4.1	Zheng et al.^53^	https://doi.org/10.1038/ncomms14049
Seurat (Seurat_4.3.1)		https://doi.org/10.32614/CRAN.package.Seurat
Tidyverse (tidyverse_2.0.0)		https://doi.org/10.21105/joss.01686
factoextra (factoextra_1.0.7)		https://doi.org/10.32614/CRAN.package.factoextra
rcna (rcna 0.0.99)		https://doi.org/10.1101/2021.04.19.440534
fgsea (fgsea 1.28.0)		https://doi.org/10.1101/060012
msigdb (msigbr_7.5.1)		https://doi.org/10.32614/CRAN.package.msigdb
escape 1.10.0		https://doi.org/10.1038/s42003-020-01625-6
Enrich Plot (enrichplot_1.22.0)		https://doi.org/10.18129/B9.bioc.enrich plot
Clusterprofiler		https://doi.org/10.1016/j.xinn.2021.100141

### Experimental model and study participant details

The study was approved by the Internal Review Board. This was not a clinical trial. Informed consent was obtained from all subjects. Twenty-three patients are included in this study. Patients were diagnosed and clinically phenotyped by fellowship-trained uveitis specialists according to standard criteria.^54^^,^^55^^,^^56^^,^^57^^,^^58^^,^^59^^,^^60^^,^^61^^,^^62^^,^^63^ Clinical features are detailed in Table S1.

### Method details

#### Collection of biospecimens

Approximately 100-250uL aqueous fluid was aspirated from the anterior chamber of the eye and 5-20 mL blood was collected by venipuncture. Two patients had two separate aqueous and blood samples collected at recurrent clinical disease flares. Aqueous and peripheral blood mononuclear cells (PBMCs) were cryopreserved in a solution of fetal bovine serum (FBS) and 10% dimethyl sulfoxide (DMSO) and stored at -80 degrees Celsius. This cohort includes four patients previously analyzed by our group^15^ and was also used in a non-overlapping analysis of macrophage chemokine signaling networks.^64^

#### Single-cell RNA sequencing

Frozen cells were thawed and washed with FBS, or 10% Roswell Park Memorial Institute Media (RPMI) followed by PBS with 0.1% bovine serum albumin. Viability was greater than 95% by trypan blue exclusion. 5′ gene expression complementary DNA (cDNA) libraries were generated using the Chromium Controller (10x Genomics) platform and sequenced on the NovaSeq Sequencing System (Illumina) by the McDonnell Genome Institute (Washington University in St. Louis, St. Louis, MO). Gene expression data are deposited at GEO: GSE229166 and V(D)J data are deposited at GEO: GSE274471.

### Quantification and statistical analysis

#### Single-cell RNA expression analysis

Sequencing reads were aligned to the human genome using Cell Ranger version 4.1. Downstream analyses of the aligned reads of the single cell RNA sequenced data (scRNA) were carried out with a commonly used R package, Seurat 4.3.0.1. Quality control cutoffs were applied to include only cells containing at least 500 unique genes and genes present in at least 70 cells. Cells with more than 11% or less than 1% mitochondrial gene content were excluded from any downstream analysis. The cells that passed quality control were normalized and scaled using SCTransform. Using runPCA and runUMAP function, the cells were dimensionally reduced. Neighborhood embedding was calculated using K Nearest Neighbors (KNN) algorithm with the FindNeighbors function and clusters created using FindClusters.

For the lineage assignment of the immune cells, clusters of cells were assigned to cell types based on the expression of CD79A (B cells), CD3D (T cells), LYZ (myeloid cells), and NCR1 (NK cells). For even more specificity, cells were given a secondary cell type based on their clusters' differential gene expression of specialized canonical gene sets. Differential gene expression through FindMarkers function of Seurat allows for a deeper look at the driving genes within each of the clusters. Within the FindMarkers function, we used the log2 fold change threshold of 1 and the minimum cell inclusion percentage of 20% to yield cluster defining genes which allow us to label the cells with an appropriate cell type. For lineage-specific analysis, immune cells classified as B cells, T cells, NK cells, or myeloid cells were separated for subsequent PCA, UMAP, and clustering. We use FindMarkers function within these specific subset analysis with the following thresholds: log2 fold change threshold of 0.25 and minimum cell inclusion percentage of 10%.

#### Removal of doublets

B cells, T cells, NK cells, and Myeloid cells were defined as above. Quality control for doublets uses the previously mentioned cell types to find cells that confidently classify as two or more cell types within our doublet removal method. A gene-expression score for each cell was calculated to assess the likelihood of belonging to each cell type. Cells scoring in the top 10% for two or more cell type categories were labeled as multiplets and removed from differential gene expression analyses.

#### GSEA and clusterprofiler

Gene set enrichment analysis (GSEA) is the comparison of a ranked gene vector to a database of gene sets that describe specific pathways and/or biological processes. We used the Hallmark, Gene Ontology (GO), and KEGG gene sets from the msigdb gene set database. The gene vectors that result from differential gene expression using FindMarker function or by correlation values from RCNA were utilized to perform the GSEA with the package fgsea and clusterprofiler. The package "enrich plot" within clusterprofiler and plotGseaTable function was used to visualize the data.

For single cell GSEA, the escape package was used with a rank-ordered list of genes expressed in each cell and the msigdb gene sets.

#### T-cell and TCR analysis [B-cell and BCR]

T-cell and B-cell enrichment libraries were generated with the Chromium Single Cell V(D)J Enrichment Kit (10x Genomics). These T-cell receptor (TCR) and B-cell receptor (BCR) libraries were loaded into the Seurat R environment. Using the scRNA Seurat object, the TCR and BCR clonotypes are matched with the singlet cells. After quality control 78% of T cells identified in the gene expression dataset had corresponding TCR data.

The TCR clonotypes were grouped according to frequency of cells in each clonotype: singleton, small,^2^^,^^3^^,^^4^^,^^5^^,^^6^^,^^7^^,^^8^^,^^9^^,^^10^ medium,^11^^,^^12^^,^^13^^,^^14^^,^^15^^,^^16^^,^^17^^,^^18^^,^^19^^,^^20^ large (≥20). BCR clonotypes are similarly grouped to TCR clonotypes, but using fewer groups such as: singleton, small,^2^^,^^3^^,^^4^^,^^5^^,^^6^^,^^7^^,^^8^^,^^9^ large (≥10). Similar clonotypes were searched for between samples via comparison of CDR3 regions; very few were found. We produced Gini coefficient for clonotype frequency for each subject as follows:

For a population of n clonotypes within a subject with ordered count values(y)

#### Principal component analysis of meta features

Principal component analysis (PCA) was performed by the factoextra R package to determine the relationships between features. These features are listed in the supplemental information. To confirm that these observations and PCs were not due to random effect, a Monte Carlo simulation was performed using 10,000 iterations and the resulting near-normal distributions were used to determine if our actual PCs were nonrandom.

#### Covarying neighborhood analysis (CNA)

Covarying neighborhood analysis (CNA) was used to determine the effect of HLA-B27 phenotype. We down-sampled our data to remove replicate sampling events of the same patients and re-ran the Seurat process of normalizing, scaling, PCA, UMAP, and neighborhood clustering. CNA uses the Seurat neighborhoods to calculate interrelatedness. Within RCNA r package version 0.0.99, an association test was run between the cell neighborhoods of all samples and the phenotype HLA-B27. This test calculated a neighborhood for each cell such that other cells within a graphical space were a specific random walk distance away. Cells within that distance are assigned membership to that neighborhood. Finally, a neighborhood abundance matrix (NAM) is calculated, which contains the percentage inclusion of each sample within each cell neighborhood. Through the application of principal component analysis on the NAM, neighborhoods were defined by their abundance changes across the samples, resulting in NAM-PCs. Finally, a test for association is performed on these NAM-PCs resulting in a correlation value for each cell neighborhood with respect to HLA-B27. The resulting vector was multiplied by the expression matrix to calculate the impact of each gene on NAM-PCs. GSEA was conducted as described above on this ordered vector.

#### Statistical analysis

Wilcox test was used within the FindMarkers function for comparison between Seurat clusters and cell types within Figure 1, Gini coefficients were used to describe the skewness and the kurtosis of the distribution of clonotype frequencies in Figure 1D. For comparison among clusters A, B, C as defined in Figure 1E, Ordinary one-way ANOVA with Tukey multiple comparison test was used for statistical analysis, with p value > 0.05 denoted as ns, p value ≤ 0.05 as ∗, p value ≤ 0.01 as ∗∗, and p value ≤ 0.001 as ∗∗∗, and p value ≤ 0.0001 as ∗∗∗∗. For comparison between anterior uveitis and posterior uveitis in Figures 3 and S3, unpaired t test with Welch’s correction was used for statistical analysis, with p value > 0.05 denoted as ns, p value ≤ 0.05 as ∗, p value ≤ 0.01 as ∗∗, and p value ≤ 0.001 as ∗∗∗, and p value ≤ 0.0001 as ∗∗∗∗. Simple linear regression was used for per-patient correlation analyses.

To avoid the bias associated with low cell numbers, we excluded samples with very low cell numbers from per-sample based comparative analyses. For the Gini coefficient analysis in Figure S1, UV165, UV186, UV221 and UV132 were excluded entirely because they contained fewer than 50 CD4 T cells/sample. For the remaining samples, CD8 gini scores from samples with < 50 CD8 T cells, and the B cell gini scores from samples with < 50 B cells were annotated as “TLTC” (too low to calculate, see Figure S1G). For analyses of antigen activated CD4 or CD8 T cells where each patient sample counted as one data point, samples with < 50 antigen activated CD4 or CD8 T cells, including UV132, UV165, UV186 for the CD4 analyses, and UV019, UV132, UV165, UV221 for the CD8 analyses, were excluded due to low cell number. For analyses of antigen activated T cells with CD4 and CD8 T cells combined where each patient sample counted as one data point, samples with < 50 antigen-activated CD4 or CD8 T cells, including UV019, UV165, UV186, and UV221, were excluded due to low cell number.
