# Supplementary material for: Compositional variation in eye-infiltrating immune cells distinguishes human uveitis subtypes
Source: iScience. 2025 Jan 30;28(3):111928. doi: 10.1016/j.isci.2025.111928 (PMC11889669; doi:10.1016/j.isci.2025.111928)
Supplement: Document S1. Figures S1–S4 and Tables S1 and S2 [file mmc1.pdf]

iScience, Volume *28*

Supplemental information

Compositional variation in eye-infiltrating immune cells distinguishes human uveitis subtypes

Christian Concepcion, Yu Xia, Yulia Korshunova, Gregory W. Bligard, Amal Taylor, Michael A. Paley, Philip A. Ruzycki, and Lynn M. Hassman

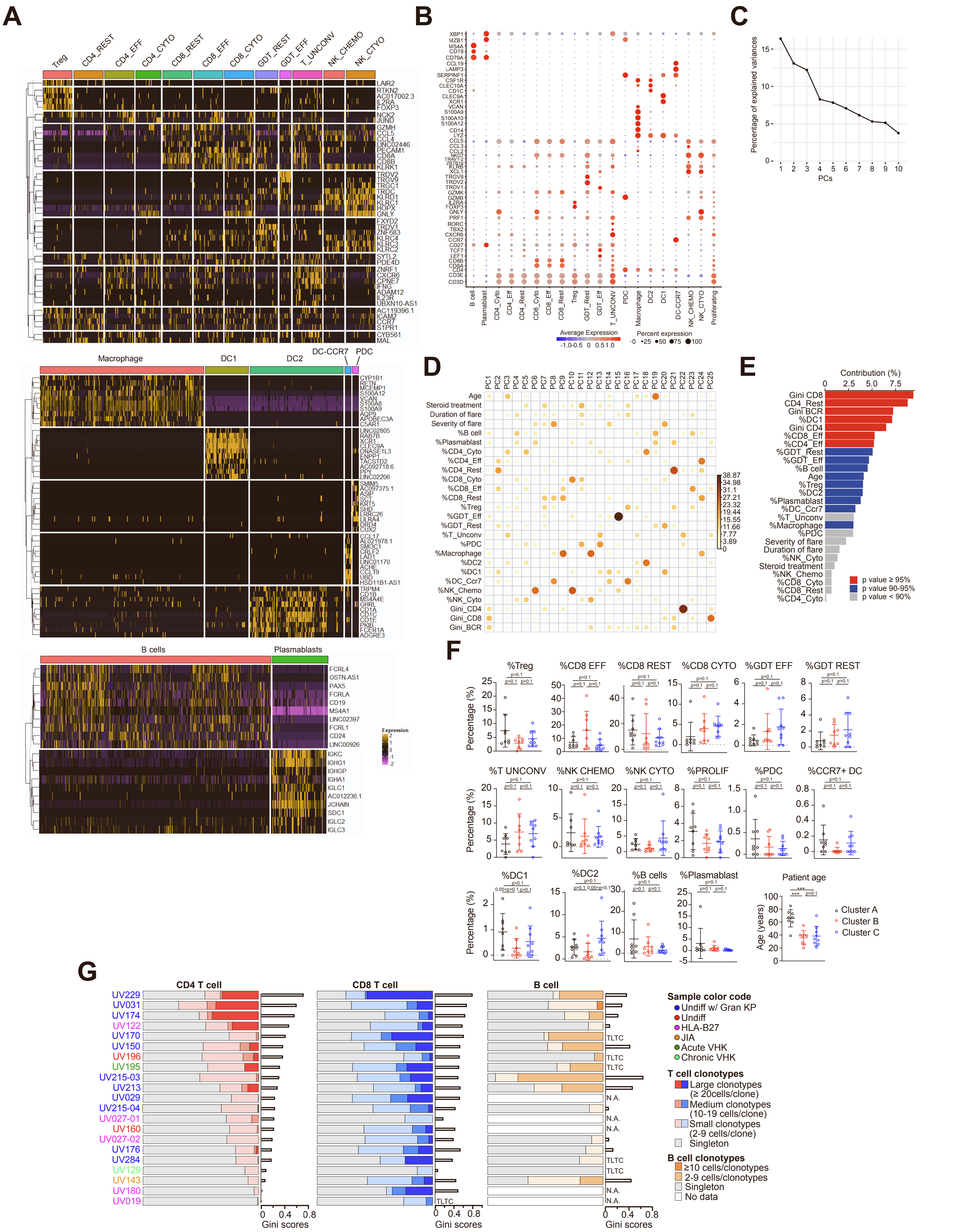

A
Treg
CD4_REST
CD4_EFF
CD4_CYTO
CD8_REST
CD8_EFF
CD8_CYTO
GDT_REST
GDT_EFF
T_UNCONV
NK_CHEMO
NK_CYTO
LAIR2
RTKN2
AC017002.3
IL2RA
FOXP3
NCK2
JUND
GZMH
CCL5
CCL4
LINC02446
PECAM1
CD8A
CD8B
KLRK1
TRDV2
TRGV9
TRGC1
TRDC
KLRD1
KLRC1
HOPX
GNLY
FXYD2
TRDV1
ZNF683
KLRC4
KLRC3
KLRC2
SYTL2
PDE4D
ZNRF1
CXCR6
CPNE7
IFNG
ADAM12
IL23R
UBXN10-AS1
AC119396.1
ICAM2
CCR7
S1PR1
CYB561
MAL
Macrophage
DC1
DC2
DC-CCR7
PDC
B cells
Plasmablasts
FCRL4
OSTN-AS1
PAX5
FCRLA
CD19
MS4A1
LINC02397
FCRL1
CD24
LINC00926
IGKC
IGHG1
IGHGP
IGHA1
IGLC1
AC012236.1
JCHAIN
SDC1
IGLC2
IGLC3
Expression
B
B cell
Plasmablast
CD4_Cyto
CD4_Eff
CD4_Rest
CD8_Cyto
CD8_Eff
CD8_Rest
Treg
GDT_Rest
GDT_Eff
T_UNCONV
PDC
Macrophage
DC2
DC1
DC-CCR7
NK_CHEMO
NK_CYTO
Proliferating
Average Expression
Percent expression
C
Percentage of explained variances
PCs
D
Age
Steroid treatment
Duration of flare
Severity of flare
%B cell
%Plasmablast
%CD4_Cyto
%CD4_Eff
%CD4_Rest
%CD8_Cyto
%CD8_Eff
%CD8_Rest
%Treg
%GDT_Eff
%GDT_Rest
%T_Unconv
%PDC
%Macrophage
%DC2
%DC1
%DC_Ccr7
%NK_Chemo
%NK_Cyto
Gini_CD4
Gini_CD8
Gini_BCR
E
Contribution (%)
Gini CD8
CD4_Rest
Gini BCR
%DC1
Gini CD4
%CD8_Eff
%CD4_Eff
%GDT_Rest
%GDT_Eff
%B cell
Age
%Treg
%DC2
%Plasmablast
%DC_Ccr7
%T_Unconv
%Macrophage
%PDC
Severity of flare
Duration of flare
%NK_Cyto
Steroid treatment
%NK_Chemo
%CD8_Cyto
%CD8_Rest
%CD4_Cyto
p value ≥ 95%
p value 90-95%
p value < 90%
F
%Treg
%CD8 EFF
%CD8 REST
%CD8 CYTO
%GDT EFF
%GDT REST
%T UNCONV
%NK CHEMO
%NK CYTO
%PROLIF
%PDC
%CCR7+ DC
%DC1
%DC2
%B cells
%Plasmablast
Patient age
Percentage (%)
Age (years)
Cluster A
Cluster B
Cluster C
G
CD4 T cell
CD8 T cell
B cell
UV229
UV031
UV174
UV122
UV170
UV150
UV196
UV195
UV215-03
UV213
UV029
UV215-04
UV027-01
UV160
UV027-02
UV176
UV284
UV129
UV143
UV180
UV019
Gini scores
TLTC
N.A.
Sample color code
Undiff w/ Gran KP
Undiff
HLA-B27
JIA
Acute VHK
Chronic VHK
T cell clonotypes
Large clonotypes (≥ 20cells/clone)
Medium clonotypes (10-19 cells/clone)
Small clonotypes (2-9 cells/clone)
Singleton
B cell clonotypes
≥10 cells/clonotypes
2-9 cells/clonotypes
Singleton
No data

Figure S1 The composition of immune cell types varies between clinical disease phenotypes, related to Figure 1 and Table S1 (A) Heatmaps showing scaled expression of cluster defining genes for cells (columns) in clusters from Figure 1A. top: T cells and NK cells, middle: myeloid cells, bottom: B cells. (B) Average expression (color scale) and percent expression (dot size) for canonical genes from cell clusters in Figure 1A. (C) Distribution of variability across the first 10 principal components (PCs) of the principal component analysis (PCA) as shown in Figure 1C. (D) Contribution of each variable to the first 25 PCs. Monte Carlo simulation of 10,000 iterations performed to obtain p values. (E) Cumulative contribution of each variable for variance across PC1 and PC2 combined. Colors denote level of significance according to key. (F) Distribution of cell type proportions and age across the three patient clusters as defined in Figure 1E represented as mean ± SEM. Ordinary one-way ANOVA with Tukey multiple comparison test was used for statistical analysis, with p value > 0.05 denoted as ns, p value ≤ 0.05 as *, p value ≤ 0.01 as **, and p value ≤ 0.001 as ***, and p value ≤ 0.0001 as ****. (G) Per-patient clonal expansion of CD4 T cells with corresponding Gini score (left), CD8 T cells with corresponding Gini coefficient (middle), and B cells with corresponding Gini coefficient (right), arranged from top to bottom in descending order of CD4 Gini coefficients. Increasing shades correspond to clonotype sizes. Sample names are colored according to clinical diagnosis (key in figure, same as in Figure 1). Samples with fewer than 50 cells were excluded from Gini analysis and annotated as “TLTC” (too low to calculate).

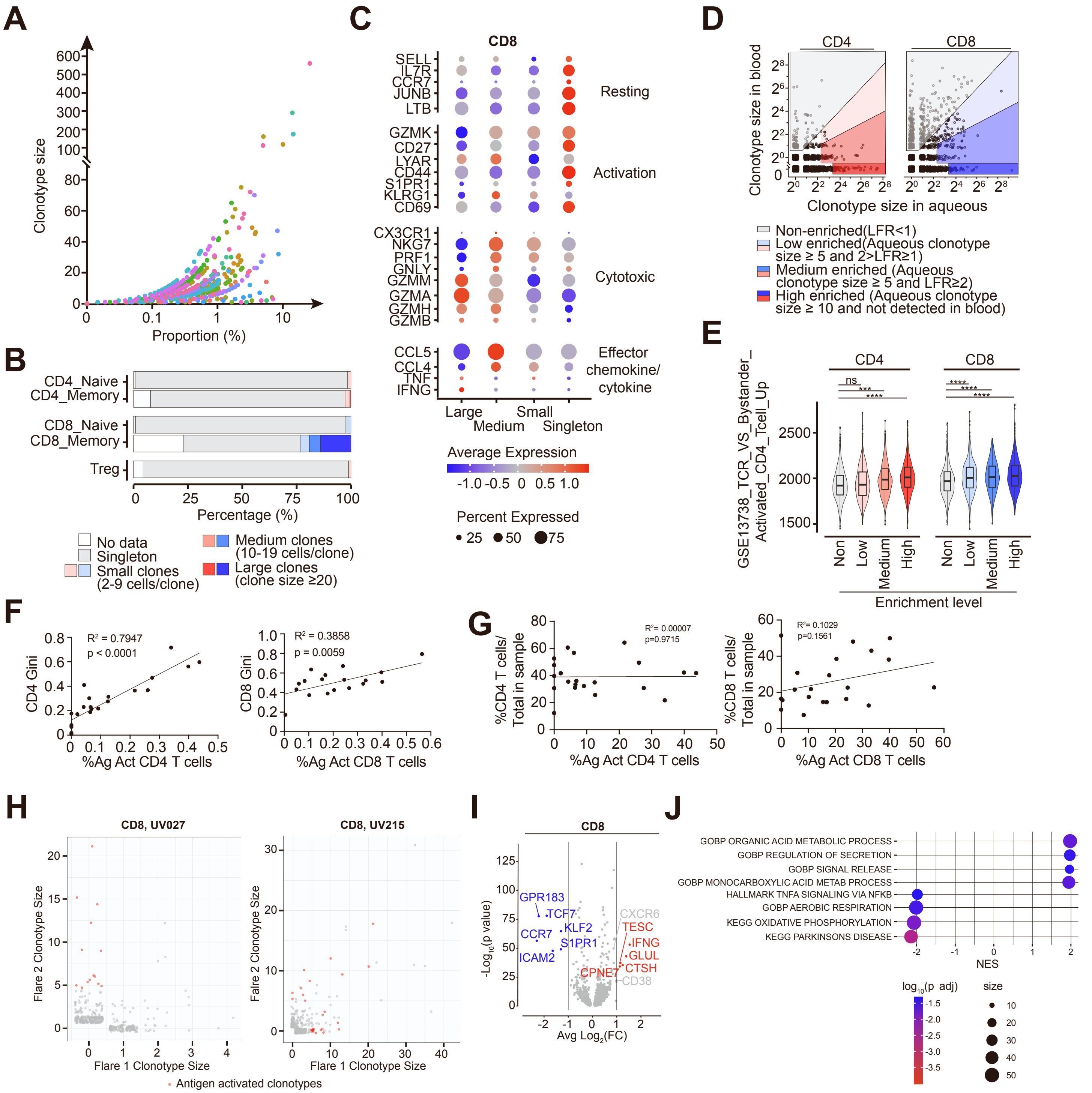

Figure S2. Ocular T cells are clonally expanded by local antigen stimulation, related to Figure 2 (A) The size of each clonotype (on the y axis) and that clonotype's proportion of total T cells (on the x axis). Each dot represents one clonotype and each color represents a unique patient. (B) Clonal expansion of each **blood** CD4 and CD8 clonotype, as shown by percent contributed by singlets (clone size = 1, gray), small clones (2-9 cells/clone, light red/blue), medium clones (10-19/clone, medium red/blue) and large clones (clone size ≥20, dark red/blue). Cells with transcriptional data but

no TCR data were marked as “no data” (white). C) Expression of selected genes in ocular CD8 T clonotype size groups (singleton, small, medium and large, as defined in panel B, color indicates relative level and circle size indicates percent express). (D) Enrichment groups based on clonotype size and relative enrichment in eye vs blood for T cells used for analysis in (E), with non-enriched log fold ratio (LFR) <1, low enriched 1=<LFR<2 and clonotype size >= 5; medium enriched = LFR>=2 and clonotype size >= 5; high enriched = detected in eye but not in blood and clonotype size >= 10, where LFR is defined as log2 (clonotype size in the eye/clonotype size in the blood) for any given clonotype. (E) Single cell gene set enrichment (GSEA) of T cell receptor signaling genes GSEA13718 in ocular CD4 (left) and CD8 (right) T cells, by enrichment group defined in D. One-way ANOVA was used for statistical analysis, with p value > 0.05 denoted as ns, p value ≤ 0.05 as *, p value ≤ 0.01 as **, and p value ≤ 0.001 as ***, and p value ≤ 0.0001 as ****. (F) Correlation between percent Ag activated T cells and Gini score for CD4 T cells (left) and CD8 T cells (right). Samples with fewer than 50 antigen-activated CD4 or CD8 T cells were excluded. Simple linear regression was carried out for statistical analysis. (G) Lack of correlation between % Ag activated CD4 T cells and CD4 T cells (left) and CD8 T cells (right) as a percentage of total aqueous T cells in each sample. Samples with fewer than 50 antigen-activated CD4 or CD8 T cells were excluded. Simple linear regression was carried out for statistical analysis. (H) Few antigen-activated CD8 clonotypes were present in both flares. Clonotype frequency in flare 1 (x-axis) vs flare 2 (y-axis) for each CD4 clonotype in two patients (eyes) that underwent serial sampling. Red indicates antigen-activated clonotypes (size >/=5 cells; LFR>1 in at least one flare) and gray indicates clonotypes that are not enriched in either flare. Patient UV027 (HLA-B27 AAU, left) and UV215 (undifferentiated uveitis with granulomatous KP, right).

(I) Differential gene expression between antigen-activated CD8 T cells (size >/=5 and LFR>1) and singleton CD8 T cells. (J) GSEA of differential gene expression between antigen-activated CD8 T cells and singleton CD8 T cells. NES normalized enrichment score, high scores are associated with antigen-activated CD8 T cells.

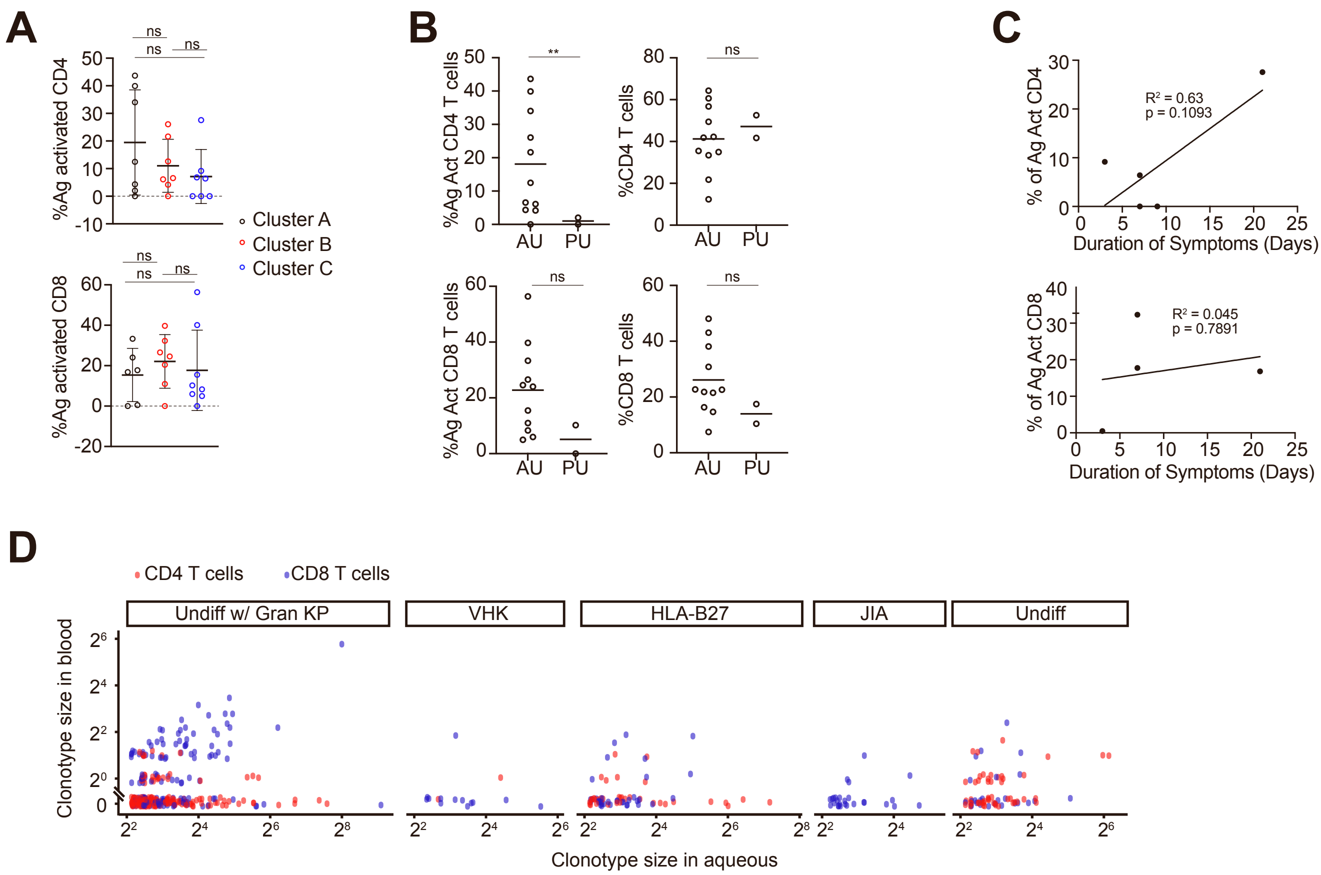

Figure S3 Antigen-activated T cells associate with key clinical features, **related to Figure 3**

(A) Percentage of antigen activated/total CD4 T cells (top) and CD8 T cells (bottom) in each cluster. The mean in each cluster is marked. Samples with fewer than 50 antigen activated CD4 or CD8 T cells were excluded. Ordinary one-way ANOVA with Tukey multiple comparison test was used for statistical analysis, with p value > 0.05 denoted as ns, p value ≤ 0.05 as *, p value ≤ 0.01 as **, and p value ≤ 0.001 as ***, and p value ≤ 0.0001 as ****. (B) Percentage of antigen activated/total CD4 T cells (top left) and CD8 T cells (bottom left), compared to percentage of all CD4 T cells/total infiltrate (top right) and CD8 T cells/total infiltrate (bottom right) for uveitis samples with anterior uveitis (AU) vs panuveitis samples with more prominent posterior inflammation (PU). Samples with fewer than 50 antigen-activated CD4 or CD8 T cells were excluded. Welch's t test was used for statistical analysis, with p value > 0.05 denoted as ns, p value ≤ 0.05 as *, p value ≤ 0.01 as **, and p value ≤ 0.001 as

, and p value ≤ 0.0001 as *. (C) Association between proportion of antigen activated CD4 (top) and CD8 (bottom) T cells with increasing duration of inflammation (in days) amongst 5 samples from patients with HLA-B27 AAU. Regression analysis was done using simple linear regression.

(D) Distribution of CD4 (red) and CD8 (blue) clonotype size in eye (x-axis) vs blood (y-axis) in select disease groups. Undiff w/ Gran KP: undifferentiated uveitis with granulomatous KP (n= 11 samples); VKH: Vogt-Koyanagi-Harada's disease (n=1); HLA-B27: HLA-B27 AAU (n=5 samples); JIA: juvenile idiopathic arthritis-associated uveitis (n=1); Undiff: undifferentiated uveitis without KP (n=3).

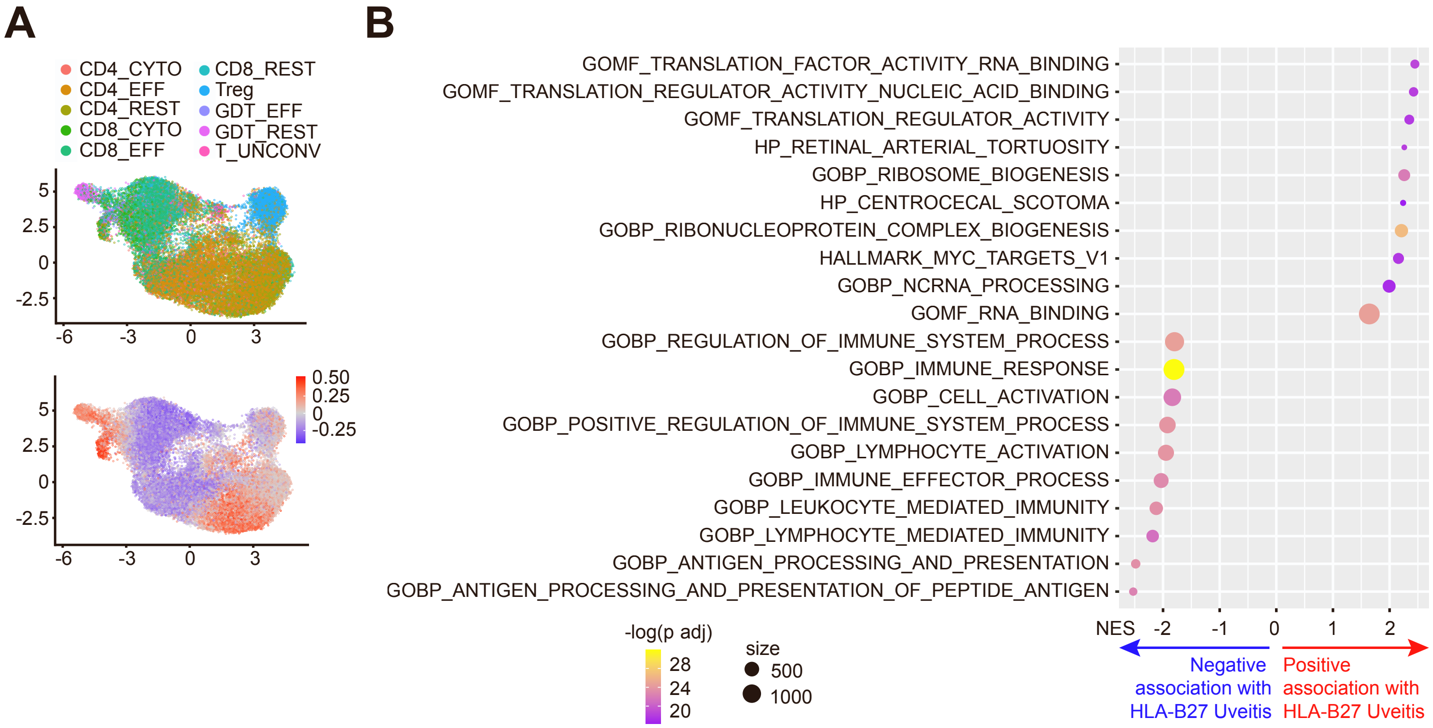

Figure S4 HLA-B27 associated acute anterior uveitis features innate activated myeloid cells, , related to Figure 4. (A) Association between ocular T cell states and HLA-B27 AAU determined by CNA; cluster identity (top) and association for HLA-B27 across all T cells (bottom), colored according to neighborhood coefficient (red: high correlation, blue: low correlation). (B) GSEA analysis of the genes driving T cell CNA in panel A.

study ID	Age	Ancestry	Gender	Phenotype	Etiology	Anatomy	Laterality	Disease course	Length of symptoms (days)	AC cell grade(SUN)	Local Therapy at sampling	Systemic Therapy at sampling	Last treatment regimen	Disease controlled	Therapies Failed
UV029	55	Black	F	Gran	Undif	PanU	Bilateral	persistent, R	1	1+	PF BID	MTX	topical prn	Y	
UV031	67	Black	F	Gran	Undif	AU_IU	Bilateral	chronic	365	2+	PF TID	none	serial injections	N	ADA, ETN, MTX
UV150	28	Black	F	Gran	Undif	AU_IU	Bilateral	chronic	90	2+	PF BID	none	DEX, topical, ADA	N	MTX, IFX
UV170	85	Black	F	Gran	Undif	PanU	Bilateral	persistent, R	8	2+	PF QID for 2 days	none	topical prn	Y	
UV174	60	Black	F	Gran	Undif	AU_IU	Bilateral	persistent, R	30	3+	none	none	FLUC 0.59mg	Y	
UV213	73	Black	F	Gran	Undif	AU_IU	Right	persistent, R	90	1+	PF BID for 1 day	none	topical prn	Y	
UV284	62	Black	F	Gran	Undif	PanU	Bilateral	chronic	270	1+	none	none	ADA, MMF	Y	
UV229	39	Black	F	Gran	Undif	PanU	Bilateral	persistent, R	90	2+	PF BID	HCQ	ADA	Y	MMF
UV129	21	Black	F	Non_Gran	VKH	PanU	Bilateral	chronic	60	1+	PF daily	PDN 20mg, MTX, ADA	MTX	Y	
UV195	39	White	M	Gran	VKH	PanU	Bilateral	chronic	7	1+	PF TID	PDN, ADA for 1 week	ADA	Y	
UV176	75	White	M	Gran	MFCPU	PanU	Bilateral	chronic	30	3+	none	none	MTX	Y	
UV186	67	White	F	Non_Gran	BSCR	PanU	Bilateral	chronic	365	0.5+	none	none	TOCI, FLUC 0.18mg	Y	MMF
UV019	26	Black	F	Non_Gran	HLA_B27	AU	UA	acute, R	9	3+	PF 8/day for 1 day	none	ADA	Y	
UV027-01	32	Black	M	Non_Gran	HLA_B27	AU	UA	acute, R	3	2+	off PF for 2 days	off PDN for 3 days, on ADA for 1 month	LTF		
UV027-02	34	Black	M	Non_Gran	HLA_B27	AU	UA	acute, R	7	2+	none	None	LTF		
UV122	28	White	F	Non_Gran	HLA_B27	AU	UA	persistent, R	21	3+	DUR qD, PF BID	GOL for 1 month	topical, IFX	N	GOL, ADA
UV180	39	White	F	Non_Gran	HLA_B27	AU	UA	acute, R	7	4+ **	q1h PF	none	FLUC 0.59mg	Y	MMF, ADA

UV132	70	Black	M	Non_Gran	Undif	AU	Right	acute, R	n/a	3+	none	none	daily topical	Y	
UV160	51	White	M	Non_Gran	Undif	AU_IU	Bilateral	chronic	60	3+	off PF for 3 days	off MMF for 1 month	ADA, MMF	Y	MMF
UV165	45	White	F	Non_Gran	Undif	PanU	Bilateral	chronic	n/a	2+	none	off AZA and ADA for 1 month	FLUC 0.59mg	Y	
UV196	26	White	M	Non_Gran	Undif	AU_IU	Bilateral	persistent, 1	7	2+	PF QID for 1 day	none	none	Y	
UV215-03	40	White	F	Gran	Undif	AU_IU	Bilateral	persistent, R	7	2+	PF BID	none	TOCI, MMF	P	ADA, MTX, TOCI
UV215-04	40	White	F	Gran	Undif	AU_IU	Bilateral	persistent, R	60	2+	PF daily	MTX	TOCI, MMF	P	ADA, MTX, TOCI
UV143	20	White	F	Non_Gran	JIA	AU	Bilateral	chronic	n/a	2+	PF QID	MTX, off ADA for 1 week	MTX, CTZ	Y	ADA, MTX
UV221	39	White	M	Non_Gran	HSV	AU	Left	persistent, R	n/a	n/a	PF BID	valacyclovir and PF	valacyclovir, topical prn	Y	

Table 1S. Demographics and Clinical features , related to Figure 1

Legend: M male**; F** female**; Gran** granulomatous**; Non_Gran** non-granulomatous; **Undif** undifferentiated**; MFCPU** multifocal chororoiditis and panuveitis**; VKH** Vogt-Koyanai-Harada's disease**; BSCR** Birdshot chorioretinitis**; HLA_B27** HLA-B27-associated acute anterior uveitis**; AU** anterior uveitis**; AU_IU** anterior and intermediate uveitis**; PanU** panuveitis**; UA** unilateral flares, but alternating eyes **R** recurrent**; 1** single episode**; *** scaled according to SUN criteria for grading anterior chamber inflammation**; **** hypopyon present; **PF** prednisolone acetate**; BID** twice daily**; TID** thrice daily**; QID** 4 times daily**; DUR** difluprednate**; q1h** every hour; **MTX** methotrexate**; HCQ** hydroxychloroquine**; PDN** prednisone**; ADA** adalimumab**; GOL** golimumab**; MMF** mycophenolate**; AZA** azathioprine**;** prn as needed**; DEX** dexamethasone implant; **FLUC** fluocinolone implant**; TOCI** tocilizumab**; LTF** lost to follow-up**; ETN** etancercept**; IFX** infliximab

	UV019	UV027-01	UV027-02	UV029	UV031	UV122	UV129	UV132	UV143	UV150	UV160	UV165	UV170	UV174	UV176	UV180	UV186	UV195	UV196	UV213	UV215-03	UV215-04	UV221	UV229	UV284
AGE	26	32	34	55	67	28	21	70	20	28	51	45	85	60	75	39	67	39	26	73	40	40	39	39	62
TREATMENT	0	6	0	2	3	4	1	0	4	2	2	0	0	0	0	10	0	3	0	0	2	1	2	2	0
DURATION	9	3	7	1	365	21	60	0	0	90	60	0	8	30	30	7	365	7	7	90	7	60	0	90	270
SEVERITY	73	44	43	39	93	125	3365	125	25	300	39	51	29	89	142	123	6	174	208	60	98	100	20	263	19
B_cell	0.01	0.01	0.03	0.03	0.19	0.04	0.03	0.01	0.14	0.05	0.01	0.00	0.01	0.04	0.01	0.01	0.00	0.02	0.00	0.24	0.01	0.01	0.00	0.03	0.03
Plasmablast	0.00	0.00	0.00	0.00	0.03	0.01	0.01	0.00	0.04	0.01	0.00	0.00	0.00	0.01	0.00	0.00	0.00	0.01	0.00	0.19	0.01	0.00	0.00	0.01	0.00
CD4_CYTO	0.02	0.01	0.01	0.02	0.00	0.02	0.02	0.01	0.00	0.05	0.02	0.00	0.01	0.01	0.02	0.00	0.00	0.01	0.03	0.02	0.03	0.03	0.02	0.00	0.01
CD4_EFF	0.02	0.03	0.11	0.05	0.27	0.09	0.11	0.14	0.11	0.22	0.07	0.26	0.08	0.25	0.07	0.06	0.14	0.14	0.52	0.07	0.53	0.56	0.33	0.10	0.20
CD4_REST	0.27	0.29	0.19	0.26	0.14	0.20	0.27	0.11	0.02	0.23	0.27	0.01	0.26	0.17	0.44	0.41	0.13	0.10	0.10	0.26	0.01	0.01	0.04	0.12	0.21
CD8_CYTO	0.03	0.05	0.10	0.03	0.00	0.05	0.05	0.02	0.01	0.12	0.05	0.05	0.11	0.01	0.01	0.02	0.00	0.01	0.01	0.00	0.06	0.04	0.04	0.01	0.01
CD8_EFF	0.01	0.01	0.03	0.04	0.05	0.05	0.02	0.03	0.42	0.02	0.10	0.14	0.05	0.05	0.09	0.05	0.12	0.10	0.03	0.03	0.24	0.16	0.27	0.14	0.04
CD8_REST	0.05	0.09	0.16	0.35	0.11	0.05	0.09	0.04	0.05	0.09	0.24	0.03	0.22	0.16	0.01	0.06	0.39	0.39	0.04	0.11	0.01	0.01	0.04	0.07	0.13
TREG	0.07	0.02	0.02	0.02	0.03	0.04	0.03	0.02	0.04	0.06	0.07	0.03	0.13	0.04	0.20	0.10	0.03	0.01	0.04	0.03	0.03	0.03	0.00	0.07	0.07
GDT_EFF	0.05	0.05	0.02	0.01	0.01	0.01	0.02	0.00	0.00	0.01	0.01	0.00	0.00	0.01	0.00	0.02	0.00	0.02	0.01	0.00	0.00	0.00	0.05	0.02	0.00
GDT_REST	0.02	0.04	0.01	0.02	0.00	0.04	0.06	0.00	0.02	0.03	0.01	0.00	0.00	0.00	0.01	0.01	0.00	0.03	0.00	0.00	0.00	0.00	0.00	0.03	0.01
UNCON_T_CELL	0.10	0.10	0.08	0.06	0.06	0.11	0.09	0.03	0.04	0.08	0.05	0.00	0.02	0.06	0.02	0.05	0.00	0.12	0.17	0.01	0.02	0.02	0.09	0.09	0.06
PDC	0.00	0.00	0.00	0.01	0.00	0.00	0.00	0.00	0.00	0.00	0.00	0.00	0.00	0.01	0.01	0.00	0.00	0.00	0.00	0.00	0.00	0.01	0.00	0.01	0.00
Macrophage	0.27	0.20	0.12	0.01	0.04	0.11	0.06	0.51	0.00	0.01	0.05	0.15	0.02	0.06	0.04	0.09	0.01	0.01	0.01	0.00	0.00	0.06	0.00	0.13	0.03
DC2	0.05	0.01	0.02	0.02	0.02	0.04	0.07	0.04	0.05	0.01	0.01	0.14	0.01	0.04	0.03	0.04	0.06	0.00	0.00	0.00	0.01	0.01	0.04	0.03	0.02
DC1	0.00	0.00	0.01	0.01	0.01	0.02	0.01	0.00	0.00	0.00	0.00	0.00	0.02	0.01	0.01	0.00	0.00	0.00	0.00	0.00	0.01	0.00	0.00	0.01	0.00
CCR7_DC	0.00	0.00	0.00	0.00	0.00	0.00	0.00	0.00	0.00	0.00	0.00	0.00	0.00	0.00	0.00	0.00	0.00	0.00	0.00	0.00	0.00	0.00	0.00	0.00	0.01
NK_CHEMO	0.01	0.02	0.02	0.02	0.00	0.06	0.00	0.01	0.01	0.01	0.01	0.02	0.00	0.01	0.00	0.00	0.05	0.00	0.00	0.00	0.00	0.00	0.09	0.03	0.09
NK_CYTO	0.01	0.06	0.03	0.03	0.01	0.03	0.03	0.01	0.02	0.01	0.01	0.18	0.02	0.04	0.03	0.03	0.01	0.00	0.01	0.01	0.01	0.01	0.00	0.05	0.03
Gini_CD4	0.01	0.22	0.22	0.23	0.60	0.47	0.08	0.13	0.07	0.37	0.21	0.07	0.41	0.56	0.18	0.02	0.08	0.31	0.37	0.28	0.30	0.23	0.00	0.72	0.17
Gini_CD8	0.31	0.17	0.17	0.52	0.67	0.58	0.06	0.00	0.45	0.53	0.43	0.10	0.61	0.64	0.54	0.49	0.22	0.51	0.52	0.54	0.49	0.43	0.18	0.79	0.37
Gini_BCR	0.00	0.00	0.00	0.00	0.28	0.08	0.00	0.00	0.44	0.42	0.00	0.00	0.47	0.22	0.13	0.00	0.00	0.29	0.04	0.46	0.64	0.06	0.00	0.36	0.11

Table 2S. Sample details, frequency of each cell type in each sample, related to Figure 1, S1

Legend. TREATMENT steroid drop equivalents; **DURATION** length of symptomatic flare in days; **SEVERITY** cell/ul.
